# Pain Localization Shift During the Convalescence Period of Osteoporotic Vertebral Compression Fracture

**DOI:** 10.3390/geriatrics10030071

**Published:** 2025-05-24

**Authors:** Oded Hershkovich, Mojahed Sakhnini, Raphael Lotan

**Affiliations:** 1 Department of Orthopedic Surgery, Wolfson Medical Center, Holon 5822012, Israel; mojahed.sakh@gmail.com (M.S.); dr.lotan@gmail.com (R.L.); 2 Gray Faculty of Medical & Health Sciences, Tel Aviv University, Tel Aviv-Yafo 6997801, Israel

**Keywords:** vertebral compression fractures, pain, osteoporosis, lumbosacral

## Abstract

Introduction: Vertebral Compression Fractures (VCF) are the most common vertebral fractures, usually osteoporotic, with rising incidence. The natural history of VCFs-related pain remains unclear, and treatment protocols are still being evaluated, ranging from conservative to surgical. Patient-reported measures have been proven inaccurate and carry significant biases. This study examines maximal tenderness location (MTL) to palpation and percussion on physical examination during VCF healing and the postoperative period. Methods: A prospective study included 40 patients treated for VCFs per the NICE guidelines (2013) from 2019 to 2021. Treatment was either conservative (*n* = 12) or surgical (*n* − 28), Balloon Kyphoplasty (BKP). All patients’ MTL were recorded in EMR (Electronic Medical Record) on every visit. BKP was offered for severe ongoing pain after a recent, unhealed vertebral fracture despite optimal pain management, progressive fracture collapse, or lack of union. Follow-up was six months. Pain evolution was analyzed using Kaplan–Meier survival curves, Log-Rank tests, Mann–Whitney U tests, *t*-tests, and logistic regression models. A *p*-value < 0.05 was considered statistically significant. Results: 12 patients were treated conservatively, and 28 underwent BKP for T12-L2 VCFs, accounting for 75% of fractures, mostly single-level fractures. All initially suffered MTL over the VCF; BKP patients showed local VCF pain resolution after 3.5 weeks following surgery while lasting seven weeks under conservative treatment. Lumbosacral pain was more prevalent following BKP (OR = 4, *p* = 0.05) and developed earlier. Conclusions: This study is novel in relating physical examination findings to fracture age and treatment provided, suggesting that VCFs-related pain is a time-related shift from local fracture pain to lumbosacral pain. Patient-reported pain scales may not reliably distinguish between these varying pain patterns. These findings suggest that only local VCF pain should be considered for surgical treatment. Future studies evaluating VCF outcomes should address physical examination and not rely solely on patient-reported metrics.

## 1. Introduction

Vertebral compression fractures (VCF) are the most common vertebral fractures, mainly involving the thoracolumbar junctional vertebrae [1]. The most common underlying pathology of VCF is osteoporosis, and with the aging world population, VCF’s incidence is rising with a significant economic burden [2]. A recent bibliometric analysis of published literature on osteoporosis VCF found 756 publications from 1900 to 2022 and a significant increase in articles in recent years [3].

Previous studies have looked at VCF patient complaints; early studies reported common complaints of radiation to the flanks and anteriorly (66%), leg radiation (6%), and associated symptoms such as nausea and abdominal or chest pain [4]. Others have reported that radiographic VCFs may be asymptomatic or show acute pain of acute fracture that usually lasts 4–6 weeks with intense pain at the fracture site [5]. Chronic pain may also occur in patients with multiple compression fractures, height loss, and low bone density, probably due to structural changes or osteoarthritis. The greater the deformity, the greater the likelihood of pain and disability. The reduced thoracic and abdominal space due to deformity may result in decreased exercise tolerance, early satiety, and weight loss. Patients may develop sleep disorders, lowered self-esteem, depression, and reduced self-care ability [5].

Studies appraised the natural history of VCF-related pain. Klazen et al. followed VCF patients 6 and 23 months following fractures using Visual Analog Scores (VAS) and pain medications. They found significant pain reduction in the first six months following the fracture [6]. Tae-Hoon et al. used patients’ pain drawings to assess VCF pain patterns. They found three types of pain distribution in acute VCFs: over the paravertebral area, mainly localized to the midline, extending beyond the paravertebral area, and low back or lumbosacral pain remote from the VCF site [7]. They concluded that pain patterns are helpful in decision-making and treatment outcome prediction in VCFs. Haiping et al. examined the association between pain location and acute VCF fracture type. They found that pain at the fracture site is often observed in central and burst fractures, whereas pain at a non-fracture site is more common in upper and lower endplate fractures [8].

Hiromitsu et al. [9] characterized four pain behavior clusters during VCF healing: 50.8% had stable, mild pain; 21.1% started with moderate pain and quickly improved to a very low pain level; 10.9% had moderate pain that initially improved but worsened after three months, and 17.2% had persistent severe pain. This study evaluated pain level but not pain location.

Vertebral fracture pain localization over time, from acute fracture local pain to long-term non-specific pain following fracture healing, has been discussed previously by spine surgeons but has not been scientifically evaluated. The National Institute for Health and Care Excellence (NICE) guidelines for diagnosing vertebral compression fractures suitable for surgery acknowledged the importance of a physical examination of the VCF patient. They recommended thoroughly evaluating the patient’s medical history, physical examination, and imaging studies [10]. During the physical examination, the healthcare provider should assess the patient’s spine for any deformities or tenderness over the affected vertebrae. According to the NICE guidelines, local pain over the fractured vertebrae is a significant parameter in assessing a vertebral compression fracture. These findings, along with the patient’s symptoms and imaging studies, will be considered when determining the appropriate course of treatment, which may include surgery [11].

VCF treatment results are usually evaluated in the literature by patient-reported pain metrics such as self-reported VAS score, functional metrics (such as Oswestry Disability Index), and quality of life (such as SF-36). The disadvantage of self-reported measures includes their sensitivity to response style effects and personality traits that may systematically influence and distort responses to survey questions. Applying psychometric models assumes invariance across respondents and assessment periods [12,13]. Jin et al. examined the correlation between patient-reported pain and physical examination findings. They found that a poor or fair consistency narrows the application of patient-reported back pain and spinal palpation tenderness in precisely localizing VCFs. The consistency between the location of radiating pain or axial spinal percussion pain findings and VCF segments is perfect. The radiating pain and axial spinal percussion pain signs were only applicable in single or contiguous VCFs [14].

Although different aspects of VCFs have been investigated, measurement of pain localization during VCF convalescence on physical examination has never been evaluated, only pain intensity. Since this aspect of VCF-related pain localization remains unclear, this study aims to examine the clinically known paradigm of local tenderness over the fractured vertebra during the acute fracture period and changing to lumbosacral pain after the fracture has healed.

## 2. Methods

### 2.1. Study Design

The study was a single-center observational, prospective cohort study which included 40 patients treated by the Orthopedic Surgery Department for Thoracolumbar Vertebral Compression fractures during 2019–2021. The institutional review board approved the study in compliance with the principles of the Declaration of Helsinki.

### 2.2. Patient Selection

The inclusion criteria were based on the NICE (The National Institute for Health and Care Excellence) guidelines [11]; all patients underwent physical examination by palpation and percussion and were found to be suffering local pain over the fractured vertebra. The Vertebral fracture was diagnosed by at least two imaging modalities: X-rays, supine and standing, when possible, and a CT scan. CT scans were used to diagnose acute fracture characteristics: fracture lines, hematoma, lack of union, or non-unios, which were evidenced as cleft signs. When fracture appearance was not considered acute on the CT scan, an MRI or bone scan was performed.

Exclusion criteria included osteomyelitis, history of malignancy, lack of follow-up in the hospital, and mental or physical status precluding participation (such as Dementia, Aphasia, or being bedridden). Since previous clinical observations suggested lumbosacral junctional pain as a relevant painful area, fractures at L5 and sacrum were excluded from the study. Patients referred to the clinic with back pain, radiographic evidence of fracture union, and maximal tenderness location unrelated to VCF level were considered healed and excluded.

### 2.3. Treatment

Patients were treated conservatively (Analgesics, Jewett Brace, and Activity modification for two months, followed by physiotherapy and osteoporosis workup in the community) or surgically by a BKP. Surgical treatment was offered for severe ongoing pain after a recent, unhealed vertebral fracture despite optimal pain management, progressive fracture collapse, or lack of union (ascertained by a cleft sign under the vertebral endplate on CT or MRI). In our spine unit, BKP is the preferred surgical treatment of VCFs; spinal instrumentation is rarely used [15].

Six months of radiographic follow-up were performed using standing X-rays. Percussion and palpation on follow-up visits evaluated the patient’s maximal tenderness location (MTL) during a spine surgeon’s physical examination. The patients were asked whether the MTL correlated to the pain they reported. These findings were recorded in the hospital’s EMR. The same physical examination was performed and recorded throughout the follow-up.

For each patient, clinical and demographic variables were recorded at baseline, including age, gender, BMI, fracture level(s), and type of treatment administered. Pain location and intensity were evaluated longitudinally at multiple time points, categorized as (1) fracture site pain, (2) lumbosacral pain, or (3) no pain. The evolution of pain was captured using an ordinal 0–3 scale and tracked in 2-week follow-up visits.

Pain trajectories were assessed to determine resolution or transition from localized to lumbosacral pain. Follow-up continued for up to one year post-treatment. Primary outcomes included time to resolution of fracture site pain, time to emergence or resolution of lumbosacral pain, and total duration of any pain. Secondary outcomes included the likelihood of pain transition between anatomical locations and presence of pain at specific follow-up time points (e.g., 8 and 12 weeks).

### 2.4. Descriptive Statistics

Descriptive statistics were used to summarize demographic and clinical characteristics, including age, gender, BMI, fracture location, and treatment type. Continuous variables were reported as means with standard deviations, and categorical variables as frequencies and percentages. Due to the ordinal and non-normally distributed nature of pain scores over time (0–3 scale), standard parametric methods such as repeated-measures ANOVA and generalized linear models were deemed inappropriate. Instead, time-to-event (survival) analysis was employed to evaluate pain resolution patterns over time. Kaplan–Meier survival curves were generated to estimate the time to resolution of (1) fracture site pain, (2) lumbosacral pain, and (3) type of pain. Comparisons between treatment groups (conservative vs. kyphoplasty) were performed using the Log-Rank (Mantel–Cox) test. Time-to-event estimates were reported with means and 95% confidence intervals. Mann–Whitney U tests were used to compare non-parametric distributions of pain duration across treatment groups. Independent samples *t*-tests were conducted as sensitivity analyses, with Levene’s test used to assess the homogeneity of variances. Binary logistic regression models were performed to evaluate predictors of pain persistence beyond 8 or 12 weeks and examine associations between treatment type and the pain transition from the fracture site to the lumbosacral region. Odds ratios (ORs) with 95% confidence intervals were reported. Statistical significance was set at *p* < 0.05. All analyses were conducted using SPSS version 20 (IBM Corp., Armonk, NY, USA).

## 3. Results

### 3.1. Cohort

A total of 40 patients between 2019 and 2021 met the study’s inclusion criteria. A total of 12 patients were treated conservatively, while 28 underwent BKP Patient follow-up, which lasted six months, and the maximal area of pain was recorded upon percussion and palpation. The conservatively treated cohort was smaller than the BKP cohort since only admitted patients were included in this study. Patients with mild to moderate pain were discharged to community care.

### 3.2. Patient Characteristics

**Figure 1:**
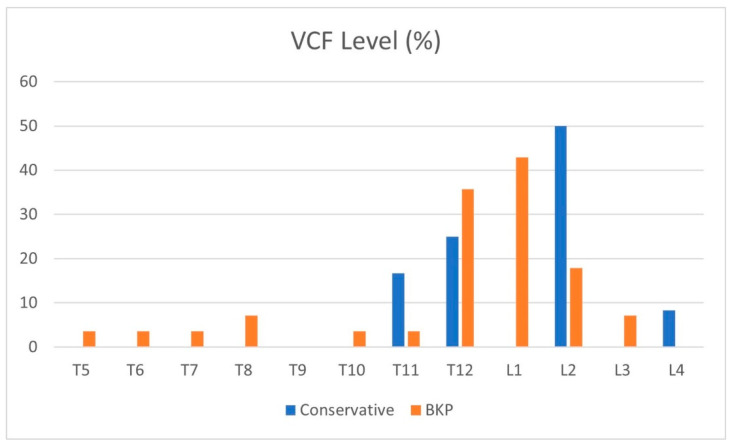
Distribution of VCF levels (%).

Patient characteristics are shown in Table 1, with an average age of 72 years in both groups (*p* = 0.93). Most patients suffered a single-level VCF; in the BKP group, five patients had two VCFs, and one patient had four VCFs, while in the conservative group, all had a single-level VCF. The average time from fracture to BKP was 29 days, and the median was 19 days. The maximal time of fracture-to-BKP was 93 and 94 days in two cases with symptomatic non-union. Figure 1 shows VCF dispersion in the thoracic and lumbar spine, with the thoracolumbar junction being the most common; T12-L2 VCFs account for 75% of the fractures treated. A single L4 VCF was included in the conservative group.

### 3.3. Pain Pattern Changes

**Figure 2:**
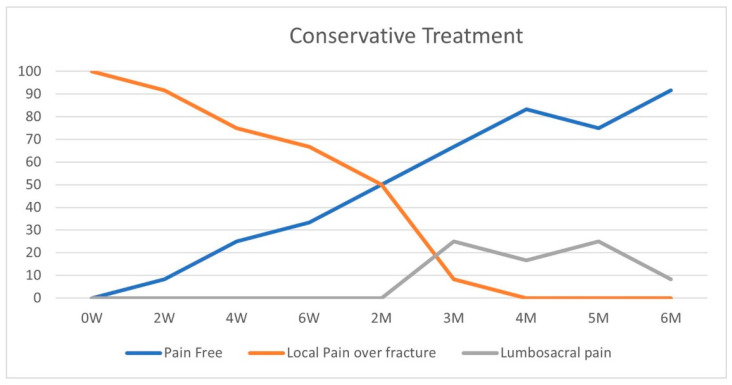
Conservative group—pain pattern over time (%).

**Figure 3:**
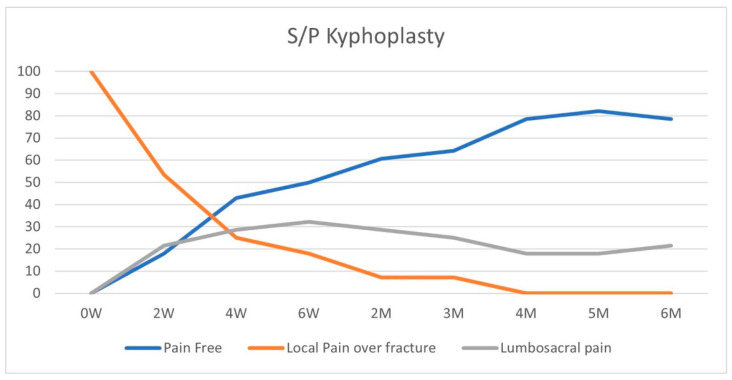
BKP treatment group—pain pattern over time (%).

On initial physical examination, patients in both groups suffered maximal tenderness over the fractured vertebrae (Figure 2 and Figure 3). In the conservative treatment cohort, fracture site MTL resolved over seven weeks on average, while in the BKP cohort, the average resolution occurred earlier, 3.5 weeks, a statistically significant difference (*p* = 0.016). A total of 23 patients out of 40 had an MTL shift, from the fractured vertebra to the lumbosacral pain, with an average of 11.7 weeks for the pain shift for the whole cohort. Only 17 patients did not develop lumbosacral pain following local fracture pain resolution; 8 of the 12 patients were treated conservatively, and 9 out of the 28 were treated with BKP.

In the conservative treatment cohort, 4 out of 12 patients had an MTL shift from local fractured vertebral MTL to lumbosacral MTL, while 19 out of the 28 BKP patients had MTL shift to lumbosacral pain, *p* = 0.043. A logistic regression for the same data also returns a significant difference between these groups, *p* < 0.05. The odds ratio for developing lumbosacral pain following a fracture treated by BKP compared to conservative treatment is 4 (*p* = 0.05; CI 1.003–17.76). The conservative treatment group had an MTL shift after an average of 15 weeks, while the BKP group shifted after nine weeks on average. The conservative group had a longer period of local fractured vertebral pain, *p* = 0.045.

Pain patterns did not differ by age or gender, *p* > 0.05. A total of 12 weeks following the fracture, BKP cohort did not have a different pain pattern dispersion compared to conservative treatment.

Examining the study’s power, a study with a 0.8 power with a medium size effect of 0.5 and one freedom degree should enroll 32 subjects. Our cohort includes 40 patients, thus exceeding this limit.

## 4. Discussion

VCFs are common, and various treatment strategies have been evaluated extensively using patients’ reported subjective pain levels and function. Patient-reported metrics have known biases; one is the expectation that patients report the exact location of their pain, compared to objective findings on physical examination by an experienced physician. Prior beliefs among spine surgeons suggested local pain over the acute fractured vertebra that may change with fracture healing. This study is novel in relating physical examination findings to fracture age and treatment, not relying on patient-reported metrics but on old-school physical examination skills.

The study included elderly patients with a mean age of 72 years who suffered VCF, as defined by the NICE guidelines, radiologically and by physical examination findings. Initially, all included patients had maximal local tenderness over the fractured vertebra on percussion or palpation, per inclusion criteria, explaining the relatively high portion of patients needing surgery and supporting the exclusion methodology.

As a rule of thumb, fracture healing takes around eight weeks [16,17], and the conservative treatment group showed 50% of pain-free patients on physical examination over this period. None of the conservatively treated patients in our study had local fracture-related pain at a four-month follow-up.

According to the NICE guidelines, we offered BKP only to patients with local pain localized to the fractured vertebra and failed conservative treatment. In the conservative treatment cohort, fracture site MTL resolved over seven weeks on average, while in the BKP cohort, the average resolution occurred earlier, 3.5 weeks, *p* = 0.016. A total of 23 patients out of 40 patients included in the study had an MTL shift from the fractured vertebra to the lumbosacral pain, with an average of 11.7 weeks for the pain shift for the whole cohort. A total of 42% of patients did not develop lumbosacral pain following local fracture pain resolution, while 58% developed lumbosacral pain. In the conservative treatment cohort, a third of the patients had an MTL shift from local fractured vertebral MTL to lumbosacral MTL, while 68% of BKP patients had MTL shift to lumbosacral pain, *p* = 0.043, odds ratio = 4. The conservative treatment group had an MTL shift after an average of 15 weeks, while the BKP group shifted after nine weeks on average. The conservative group had a longer period of local fractured vertebral pain, *p* = 0.045.

The vast majority of VCFs are treated conservatively in the community. Those who failed conservative treatment or had a suspicion of instability (significant local kyphosis, vertebral vacuum sign) reached a hospital’s spine service [15]. The results support the previous conception of a changing pain pattern following a VCF. Most patients, 58%, develop lumbosacral pain following fracture healing.

Most of our cohort comprised patients admitted for BKP, and a minority was found to be compatible with conservative treatment, leading to a possible selection bias of patients with severe ongoing pain after a recent, unhealed vertebral fracture despite optimal pain management pain, progressive fracture collapse, or non-union, thus making this cohort more pain prone with possibly worse sagittal kyphotic malalignment. Our results showed that BKP reduces local fracture pain period by 50%, from 7 weeks to 3.5 weeks, but did not prevent lumbosacral pain. In our cohort, BKP patients had four times more lumbosacral pain. Since BKPs were not performed on L5 or sacral fractures, the surgery itself cannot explain lumbosacral pain development, suggesting an indirect cause. We hypothesize that lumbosacral pain is mechanical due to the change in the lumbar spine’s sagittal alignment caused by the fracture, leading to myofascial pain or facet joint overloading.

On follow-up visits, patients with persistent pain did not localize the pain on anamnesis but reported variable levels of backache. Only upon physical examination did the patients discern the primary pain location over the fractured vertebra or lumbosacral junctional pain.

This study shows that VCF-related pain is probably not a single entity but a time-related shift from local fracture pain to lumbosacral junctional pain. The lumbosacral Junctional pain can be attributed to sagittal alignment changes with muscle fatigue or muscular disuse [18,19]. BKP reduces the VCF-related pain period, thus unveiling early lumbosacral junctional pain.

As published by Jin et al. [14], although easier to measure, patient-reported pain does not correlate to physical examination findings; therefore, it cannot be used as a single measurement to assess the VCF pain pattern. This study’s location of maximal tenderness is not intended to be used as a VCF outcome measurement but rather to give an anatomic context to the VAS, ODI, and quality-of-life measurements. This study is novel in describing the change in pain location following a VCF’s treatment, conservative or surgical.

The study’s limitations include small cohort size, observational nature, seemingly short follow-up, and lack of patient-reported metrics. This study is an observational clinical study, so it does not investigate the pathophysiological source of pain over time or radiological characteristics. Patient-reported pain scales and functions were not collected; thus, correlating pain-pattern change to overall pain/function was impossible. The follow-up period was seemingly relatively short, only six months, but long enough to assess pain location and fracture healing. This period was satisfactory as the pain diminished over time in a linear pattern. Seven patients in the BKP group had more than a single fractured vertebra, emphasizing pain reduction following BKP pain relief.

This study emphasizes the importance of physical examination in treatment decision-making, not solely relying on imaging studies. Patients who lack maximal tenderness location over the fractured vertebra probably should not be considered for surgical treatment since lumbosacral pain is a late symptom following a fracture and not a direct fracture-related pain. Despite the study’s limitations, statistically significant results were obtained, supporting a pain location difference between the two (conservative/surgical) cohorts over time. Further randomized controlled studies should be conducted with larger cohorts, including physical examination results combined with patient-reported scores, to comprehend the actual VCF pain patterns and the impact of the treatment options.

## 5. Conclusions

In conclusion, this study is novel in elucidating how pain patterns change following VCFs and are affected by the treatment provided. This study reinforces the value of adequately documenting old-fashioned physical examinations in treatment decision-making. Patients who lack maximal tenderness location over the fractured vertebra probably should not be considered for surgical treatment since lumbosacral pain is a late symptom following a fracture and not a direct fracture-related pain. Future studies will have to address the findings of this study to understand the complexity of VCF pain patterns and achieve a reliable understanding of vertebral augmentation procedures’ efficacy and role.

## Figures and Tables

**Table 1:** Cohort characteristics.

*p*-Value	BKP	Conservative	
	28	12	No. of patients
0.30	25.9%	41.7%	Males
0.93	72.2 ± 10.1	72.5 ± 13.4	Age (years)
0.90	27.3 ± 3.9	26.8 ± 2.5	BMI
0.14	1.29 (1–4)	1	Average No. of VCFs
	29 ± 26.9 (Median 19)	-	VCF to BKP Time (days)

## Data Availability

The datasets generated and/or analyzed during the current study are available from the corresponding author upon reasonable request.

## Glossary

VCF	Vertebral Compression Fracture
BKP	Balloon Kyphoplasty
LSP	Lumbosacral pain
MTL	Maximal tenderness location
